# Preclinical Evaluation of a Novel High-Affinity Radioligand [^99m^Tc]Tc-BQ0413 Targeting Prostate-Specific Membrane Antigen (PSMA)

**DOI:** 10.3390/ijms242417391

**Published:** 2023-12-12

**Authors:** Ekaterina Bezverkhniaia, Panagiotis Kanellopoulos, Ayman Abouzayed, Mariia Larkina, Maryam Oroujeni, Anzhelika Vorobyeva, Ulrika Rosenström, Vladimir Tolmachev, Anna Orlova

**Affiliations:** 1 Department of Medicinal Chemistry, Uppsala University, 751 23 Uppsala, Sweden; ekaterina.bezverkhniaia@ilk.uu.se (E.B.); panagiotis.kanellopoulos@ilk.uu.se (P.K.); ayman.abouzayed@ilk.uu.se (A.A.); ulrika.rosenstrom@ilk.uu.se (U.R.); 2 Research Centrum for Oncotheranostics, Research School of Chemistry and Applied Biomedical Sciences, Tomsk Polytechnic University, 634009 Tomsk, Russia; lms31@tpu.ru; 3 Scientific and Research Laboratory of Chemical and Pharmaceutical Research, Siberian State Medical University, 634050 Tomsk, Russia; 4 Department of Immunology, Genetics and Pathology, Uppsala University, 752 37 Uppsala, Sweden; maryam.oroujeni@igp.uu.se (M.O.); anzhelika.vorobyeva@igp.uu.se (A.V.); vladimir.tolmachev@igp.uu.se (V.T.); 5 Affibody AB, 171 65 Solna, Sweden; 6 Science for Life Laboratory, Uppsala University, 752 37 Uppsala, Sweden

**Keywords:** prostate cancer, prostate-specific membrane antigen, PSMA, technetium-99m, BQ0413, single-photon emission computed tomography

## Abstract

Radionuclide imaging using radiolabeled inhibitors of prostate-specific membrane antigen (PSMA) can be used for the staging of prostate cancer. Previously, we optimized the Glu-urea-Lys binding moiety using a linker structure containing 2-napththyl-L-alanine and L-tyrosine. We have now designed a molecule that contains mercaptoacetyl–triglutamate chelator for labeling with Tc-99m (designated as BQ0413). The purpose of this study was to evaluate the imaging properties of [^99m^Tc]Tc-BQ0413. PSMA-transfected PC3-pip cells were used to evaluate the specificity and affinity of [^99m^Tc]Tc-BQ0413 binding in vitro. PC3-pip tumor-bearing BALB/C nu/nu mice were used as an in vivo model. [^99m^Tc]Tc-BQ0413 bound specifically to PC3-pip cells with an affinity of 33 ± 15 pM. In tumor-bearing mice, the tumor uptake of [^99m^Tc]Tc-BQ0413 (38 ± 6 %IA/g in PC3-pip 3 h after the injection of 40 pmol) was dependent on PSMA expression (3 ± 2 %IA/g and 0.9 ± 0.3 %IA/g in PSMA-negative PC-3 and SKOV-3 tumors, respectively). We show that both unlabeled BQ0413 and the commonly used binder PSMA-11 enable the blocking of [^99m^Tc]Tc-BQ0413 uptake in normal PSMA-expressing tissues without blocking the uptake in tumors. This resulted in an appreciable increase in tumor-to-organ ratios. At the same injected mass (5 nmol), the use of BQ0413 was more efficient in suppressing renal uptake than the use of PSMA-11. In conclusion, [^99m^Tc]Tc-BQ0413 is a promising probe for the visualization of PSMA-positive lesions using single-photon emission computed tomography (SPECT).

## 1. Introduction

The incidence of prostate cancer is substantial, ranking it second highest malignancy diagnosis after lung cancer made in men and the fifth leading cause of death worldwide. In 2018, 1.2 million new cases of prostate cancer were registered all over the world, representing 7.1% of all cancers among men. When prostate cancer is suspected, tissue biopsy remains the standard diagnostic method; however, it can miss 21% to 28% of prostate cancers and undergrade 14% to 17% [1]. The discerning and characterization of this malignancy have become increasingly precise because of advances in magnetic resonance and functional imaging, as well as the emergence of specific biomarkers. Interest has grown in molecular imaging using positron emission tomography (PET) and single-photon emission computed tomography (SPECT) for the diagnosis of prostate cancer [2]. Several radiolabeled imaging probes have been approved for the visualization of prostate cancer [3]. Among the cellular mechanisms and molecular targets that can be used for diagnostic imaging in prostate cancer patients are cell division, including upregulated metabolic activity and the synthesis of cell components; osteoblastic activity in bone metastases; and numerous extracellular antigens and receptors, e.g., androgen receptor, prostate-specific membrane antigen (PSMA), gastrin-releasing peptide receptor (GRPR), urokinase-type plasminogen activator (uPA), prostate stem cell antigen (PSCA), six transmembrane epithelial antigen of prostate (STEAP1), and CD46 [4].

One of the molecular targets used for the visualization of prostate cancer is PSMA (also known as glutamate carboxypeptidase II, folate hydrolase 1, N-acetylated *α*-linked acidic dipeptidase, or N-acetyl-L-aspartyl-L-glutamate peptidase). PSMA is a type II transmembrane protein containing 750 amino acids (707 extracellular, 24 transmembrane, and 19 intracellular amino acids). In the past two decades, extensive efforts have been made to develop agents targeting PSMA for prostate cancer imaging and therapy [5]. PSMA expression in prostate cancer can be 100- to 1000-fold higher than in normal tissues. Overexpression can also be observed in cancerous lymph nodes and bone metastases. PSMA is expressed in a very high proportion of prostate cancer tumors and at nearly all stages of the disease. The transmembrane conformational structure of PSMA allows it to internalize agents bound to it, which is a highly attractive feature for targeted therapy using radiometals [6].

The first imaging probe for imaging PSMA was the full-length antibody capromab pendetide, labeled with indium-111 for SPECT [7]. However, capromab pendetide, as well as later-developed PSMA-targeting antibodies, demonstrated low imaging sensitivity, and the focus on the development of PSMA imaging probes was shifted toward small pseudo-peptide-based probes. Compared with anti-PSMA antibodies, small-molecule PSMA-targeting ligands have demonstrated better contrast and reduced radiation exposure [8]. Several small-molecule PSMA inhibitors with advantages such as rapid extravasation, rapid diffusion in the extravascular space, and effective blood clearance have been developed [9]. These small-molecule inhibitors are dominant over peptide inhibitors in the development of agents for theranostics targeting PSMA. Three types of small-molecule PSMA inhibitors have been reported: phosphorus-based, thiol-based, and urea-based ligands. The most common and extensively investigated type of high-affinity inhibitors are urea-based ligands containing a Glu-urea-Lys binding moiety [10]. To date, three small-molecule PSMA inhibitors from this group, [^68^Ga]Ga-PSMA-11, [^18^F]F-DCFPyL, and Flotufolastat F-18, have been approved for PET visualizations of prostate cancer by the Food and Drug Administration; [^18^F]F-PSMA-1007 is awaiting regulatory approval in European countries pending a phase 3 clinical study being completed; and [^99m^Tc]Tc-MIP-1404 has been reported to be a promising SPECT agent [11,12]. The most prominent ligands for PSMA-targeted endoradiotherapy using beta- and alpha-emitters are today PSMA I&T and PSMA-617 containing a DOTA chelator [9].

For the development of novel and more efficacious PSMA-targeting agents with enhanced tumor uptake and minimized off-target uptake, many structural determinants need to be considered in the molecular design. The charge of the agent; the length of the linker between the PSMA ligand and the radionuclide; and its chemical composition, hydrophilicity, or lipophilicity can be investigated. In the past two decades, tremendous efforts have been undertaken to optimize small-molecule inhibitors for PSMA-targeted imaging and therapy development [10].

PSMA ligands may be used for single-photon-emission computed tomography (SPECT), PET/MRI, and PET/CT, but most studies have utilized PSMA-PET/CT. Compared with SPECT, PET technology provides better spatial resolution; less attenuation (because of higher photon energy) and fewer scatter artifacts; and, consequently, superior diagnostic capabilities. These advantages, however, come with a high-cost burden that limits the availability of PET imaging. Most positron-emitting radioisotopes have short half-lives and require in-house cyclotron production. Therein lies the main advantage of SPECT. Radiopharmaceuticals used for SPECT imaging are cheaper and easy to distribute [13]. In Europe, hospitals have 3.5 times more SPECT than PET cameras [14]. In addition, advances in SPECT technology, such as the introduction of cadmium–zinc–telluride (CZT) SPECT cameras, have significantly improved spatial resolution and sensitivity and provided accurate quantifications of the tracer’s uptake, which is comparable to the accuracy of PET/CT [15]. Thus, the development of radiopharmaceuticals for the SPECT imaging of PSMA might improve the availability of such diagnostics.

**Figure 1:**
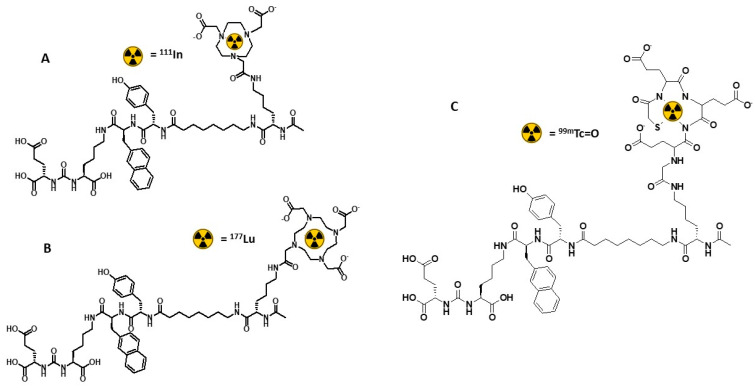
Chemical structures of [^111^In]In-BQ7859 (**A**), [^177^Lu]Lu-BQ7876 (**B**), and [^99m^Tc]Tc-BQ0413 (**C**).

Earlier, we performed an optimization of a linker between the Glu-urea-Lys binding moiety and a chelator. A linker structure of BQ7859 containing 2-napththyl-L-alanine and L-tyrosine has been found to be the optimal variant. In vivo evaluation has demonstrated that [^111^In]In-BQ7859, which includes this linker and NOTA chelator (Figure 1A), is a promising radioligand that provides efficient targeting of PSMA-expressing xenografts in mice [16]. Furthermore, BQ7876, a ligand containing the same linker and DOTA chelator (Figure 1B), was successfully labeled with ^177^Lu for radionuclide therapy and demonstrated a favorable biodistribution and dosimetry in a preclinical model [17]. [^111^In]In-BQ7859 can also be used for SPECT imaging. However, ^99m^Tc (T_1/2_ = 6 h) is a more attractive alternative because of the better imaging properties and better logistics aspects of its application.

Tc-99m is the most frequently used SPECT radionuclide. The optimal energy of emitted photons for imaging, its wide availability, and the low costs of ^99^Mo/^99m^Tc generators have facilitated its frequent use in diagnostic imaging [13]. Therefore, several PSMA-targeting radiopharmaceuticals (PSMA I&S, PSMA-T4, and MIP-1404) have been developed for radiolabeling with Tc-99m to provide simply prepared radiotracers for clinical use [9,18]. These tracers contain different chelators for coupling Tc-99m, maG_3_ (mercaptoacetyl–triglycine), HYNIC (6-hydrazinonicotinic acid), and CIM (2,2′-(2,2′-(azanediylbis(methylene))bis(1H-imidazole-2,1-diyl))diacetic acid). The selection of an optimal chelator is essential for successful clinical translation because chelators influence the uptake of PSMA ligands in normal tissues [11]. Mercaptoacetyl-containing peptide-based chelators for labeling with Tc-99m offer a number of advantages. These chelators enable the coupling of Tc-99m to the targeting moiety, which is stable in vivo [19,20]. The use of such chelators permits the formulation of a single-vial kit for the labeling procedure, which simplifies translation into clinics. The utility of a mercaptoacetyl-containing chelator, maS_3_, for labeling PSMA ligands has been confirmed in clinical evaluations of ^99m^Tc-PSMA I&S [19,20]. An attractive feature of a mercaptoacetyl-containing peptide-based chelator is the possibility of modifying the charge and hydrophilicity of the nuclide–chelator complex (and of the whole construct), and in this way, altering its biodistribution properties. In our studies on labeling a HER2-targeting affibody, we found that the use of glutamate-containing variants enables more stable complexes of Tc-99m than when mercaptoacetyl-triglycine (maGGG) is used. The use of glutamate-based chelators (such as maEEE) instead of glycine-containing ones has resulted in conjugates with lower hepatic uptake and reduced uptake in salivary glands [21]. It has been reported that negatively charged linkers can reduce off-target uptake [10]. Therefore, we thought that incorporating glutamates into a mercaptoacetyl-containing chelator for the labeling of PSMA ligands with Tc-99m would be desirable.

Following this logic, we designed the PSMA ligand BQ0413, which contains 2-napththyl-L-alanine and L-tyrosine in the linker to improve binding to PSMA and the mercaptoacetyl–triglutamate chelator (maEEE) for labeling with Tc-99m for the diagnostic imaging of PCa using SPECT (Figure 1C).

The aim of this study was to evaluate the labeling of BQ0413 with technetium-99m and the cellular processing of [^99m^Tc]Tc-BQ0413 in PSMA-expressing cells, as well as its target specificity and binding affinity to PSMA. In vivo, biodistribution and tumor targeting were studied in mice bearing human PSMA-expressing xenografts. The biodistribution and targeting properties of [^99m^Tc]Tc-BQ0413 were compared with the PSMA-targeting agent [^68^Ga]Ga-PSMA-11. We also compared the use of the addition of BQ0413 vs. PSMA-11 for blocking the uptake of [^99m^Tc]Tc-BQ0413 in normal tissues without blocking tumor uptake as one of the concepts for improving tumor targeting and the biodistribution properties of the imaging probes.

## 2. Results

### 2.1. Radiolabeling and In Vitro Characterization of [^99m^Tc]Tc-BQ0413

BQ0413 was radiolabeled with technetium-99m with a high radiochemical yield (98.5 ± 0.5%, n = 7) determined using iTLC. The presence of technetium colloid, determined using iTLC, was not higher than 0.2–0.3%. The iTLC data were cross-validated using radio-HPLC (Figure S1 in Supplementary Material). There was no release of activity after the incubation of the radiolabeled tracer with a 300-fold molar excess of cysteine or with PBS at room temperature for 1 h and in human plasma at 37 °C for 1 h.

**Figure 2:**
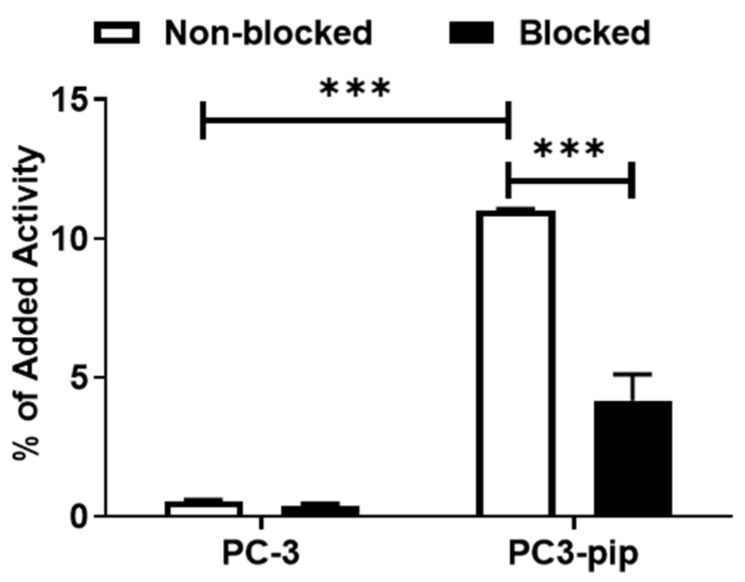
In vitro binding specificity of [^99m^Tc]Tc-BQ0413 using PSMA-transfected PC3-pip and PSMA-negative PC-3 cells. The error bars represent the standard deviation. *** indicates a *p*-value less than 0.0001 in an unpaired *t*-test.

Binding specificity was assessed in PSMA-transfected PC3-pip cells with a saturation assay and via comparison with binding to untransfected PC-3 cells. Addition of a 250-fold molar excess of PSMA-11 resulted in a significant (*p* ˂ 0.0001, unpaired *t*-test) reduction in [^99m^Tc]Tc-BQ0413 binding to PSMA-expressing PC3-pip cells. Binding to these cells was significantly higher (*p* ˂ 0.0001) than binding to PSMA-negative PC-3 cells (Figure 2).

**Figure 3:**
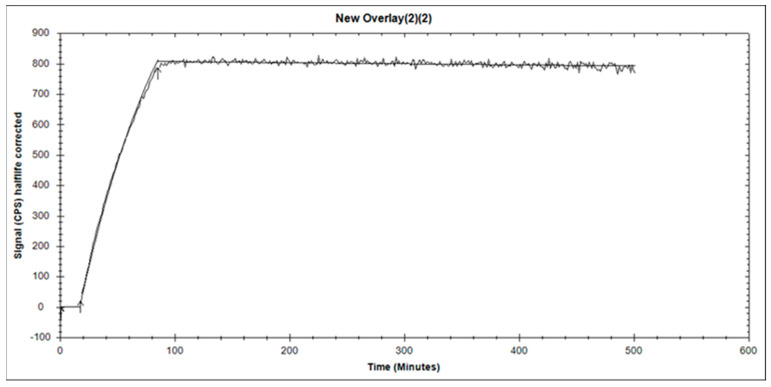
The sensorgram obtained using LigandTracer Yellow for 5 nM of [^99m^Tc]Tc-BQ0413 tested on PC3-pip cells. The arrows point the beginning and the end of incubation with radioligand. The resulted curve was fitted to a 1:1 interaction model.

The binding kinetics of [^99m^Tc]Tc-BQ0413 to PSMA were measured in real time on living PC3-pip cells, and the sensorgram is shown in Figure 3. The binding was fitted to a 1:1 interaction model. A reasonably high on-rate (4.2 ± 0.2 × 10^4^ M^−1^ × s^−1^) and a very slow off-rate (3 ± 1 × 10^−11^ M^−1^ × s^−1^) were observed. The equilibrium dissociation constant, K_D_, was 33 ± 15 pM.

**Figure 4:**
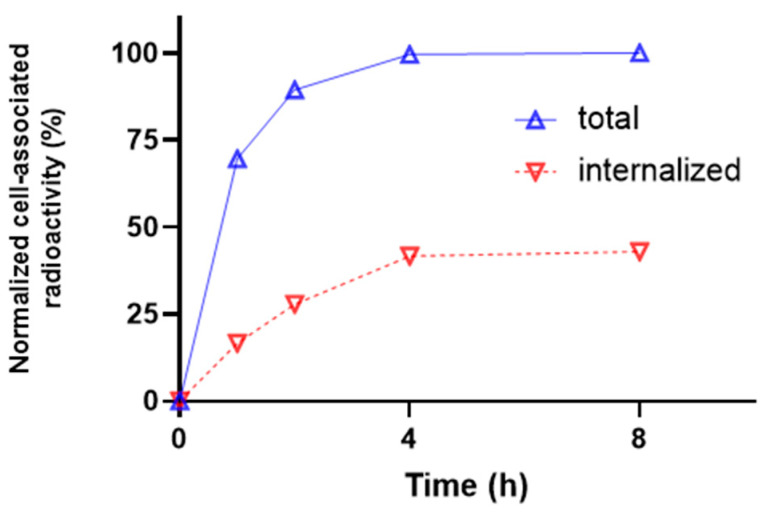
Cellular processing of [^99m^Tc]Tc-BQ0413 tested on PC3-pip cells at 1, 2, 4, and 8 h of incubation at 37 °C. The error bars (not visible because they are smaller than data point symbols) represent the standard deviation.

The cellular processing of [^99m^Tc]Tc-BQ0413 (Figure 4) showed relatively fast binding. The total cell-associated activity and internalized activity increased with time and reached a plateau after 4 h of incubation and remained at the same level of 50% cell-associated activity for another 4 h.

### 2.2. In Vivo Characterization

**Figure 5:**
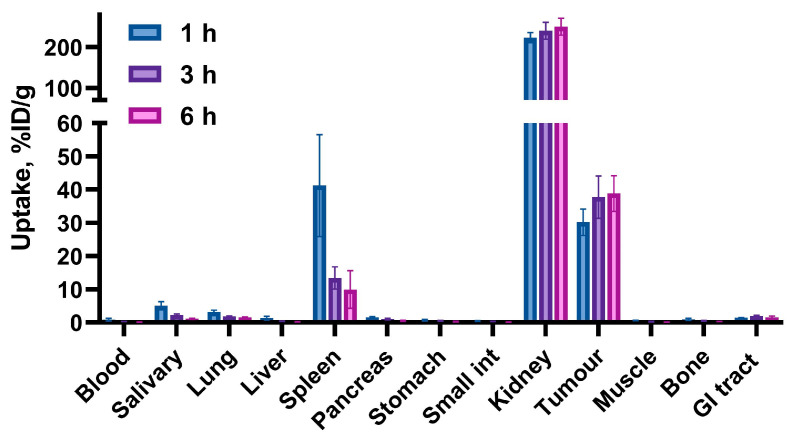
In vivo biodistribution of [^99m^Tc]Tc-BQ0413 (40 pmol/mouse) in PC3-pip tumor-bearing mice at 1, 3, and 6 h post-injection showing the uptake activity in specified organs. Activity in gastrointestinal tract (with content) is presented as %IA/sample. The data are presented as the average (n = 4) and SD.

The biodistribution profile of [^99m^Tc]Tc-BQ0413 after an injection of 40 pmol was studied at 1, 3, and 6 h pi in BALB/c nu/nu mice bearing PC3-pip xenografts. The activity uptake in the different organs is presented in Figure 5 (details in Table A1 of Appendix A). At 1 h pi, the biodistribution pattern was characterized by an elevated uptake in the kidneys (224 ± 12 %IA/g), spleen (41 ± 15 %IA/g), tumor (30 ± 4 %IA/g), salivary gland (5 ± 1 %IA/g), and lungs (3.2 ± 0.5 %IA/g). The uptake of activity in the remaining organs at 1 h pi was approximately 1 %IA/g or below. At 3 h pi, the activity uptake in the kidneys (241 ± 21 %IA/g) and tumor (38 ± 6 %IA/g) remained at the same level as 1 h pi (no significant difference; *p* > 0.05 in an unpaired *t*-test). The activity uptake decreased in other organs, which had previously demonstrated an elevated level of uptake at 1 h pi. At 6 h pi, no significant difference (*p* > 0.05 in an unpaired *t*-test) was observed regarding activity uptake in the kidneys (251 ± 21 %IA/g) and tumor (39 ± 5 %IA/g) compared with earlier time points. The level of uptake in the spleen at 6 h pi did not differ significantly from the uptake at 3 h pi. The activity uptake decreased in the rest of the organs compared to 1 and 3 h pi. The washout from the blood, liver, muscles, and bones was close to 100%.

**Figure 6:**
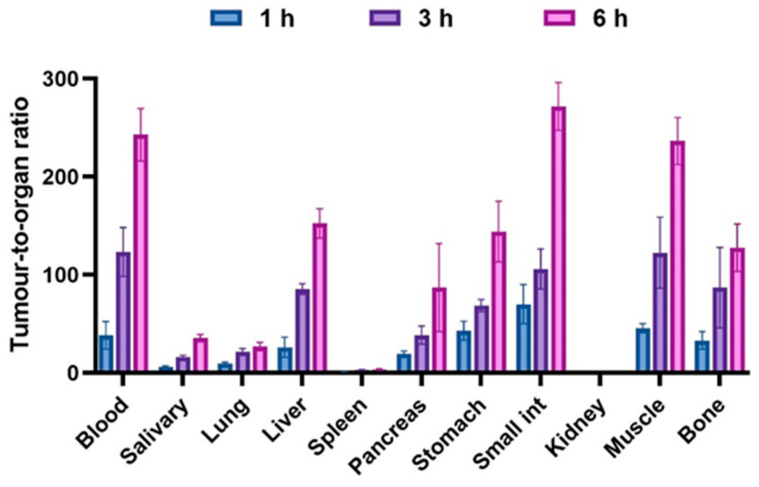
Tumor-to-organ ratios of [^99m^Tc]Tc-BQ0413 (40 pmol/mouse) in PC3-pip tumor-bearing mice at 1, 3, and 6 h pi. The data are presented as the average (n = 4) and SD.

Tumor-to-organ ratios (T/O) after an injection of 40 pmol are presented in Figure 6 (details in Table A2 of Appendix A). T/O increased from 1 to 6 h pi for all organs. The highest ratios were T/blood (243 ± 27), T/muscle (236 ± 24), T/liver (152 ± 14), and T/bone (128 ± 24) at 6 h pi.

**Figure 7:**
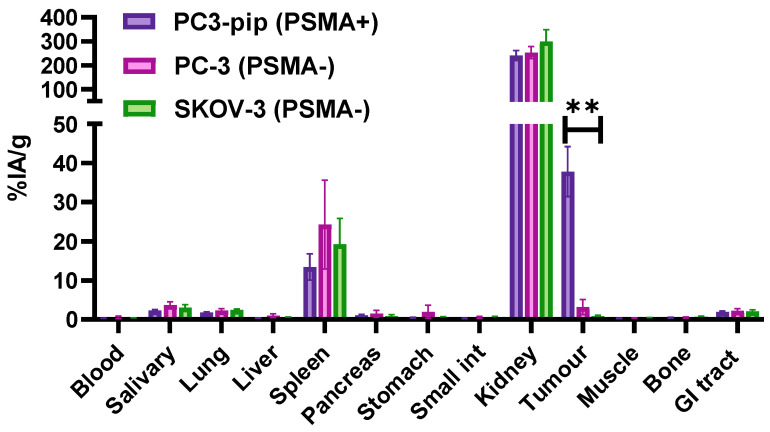
Comparison of [^99m^Tc]Tc-BQ0413 (40 pmol/mouse) accumulation in PSMA-positive PC3-pip, PSMA-negative PC-3, and SKOV-3 tumor-bearing mice 3 h pi. ** indicates a *p*-value of less than 0.05 in a one-way ANOVA test. Activity in gastrointestinal tract (with content) is presented as %IA/sample. The data are presented as the average (n = 4) and SD.

A comparison with two different PSMA-negative control xenografts, PC-3 (prostate carcinoma) and SKOV-3 (ovarian carcinoma), was performed to evaluate if the accumulation of [^99m^Tc]Tc-BQ0413 in PC3-pip xenografts was PSMA-specific. The results of this comparison are demonstrated in Figure 7 (details in Table A3 of Appendix A). The uptake of [^99m^Tc]Tc-BQ0413 in PSMA-positive PC3-pip xenografts (38 ± 6 %IA/g) was significantly (*p* < 0.05, one-way ANOVA) higher than in PSMA-negative PC-3 xenografts (3 ± 2 %IA/g) and SKOV-3 xenografts (0.9 ± 0.3 %IA/g) at 3 h pi. The tumor uptake in PC3-pip xenografts was 12.6-fold higher compared with PC-3 and 42-fold higher compared with SKOV-3.

The efficacy of the blocking of [^99m^Tc]Tc-BQ0413 uptake in normal PSMA-expressing tissues was evaluated via a co-injection of 40 pmol of [^99m^Tc]Tc-BQ0413 with 5 nmol/animal of unlabeled BQ0413 or 5 nmol/animal of the unlabeled PSMA-analog PSMA-11. Co-injected animals demonstrated significantly (*p* < 0.05, one-way ANOVA) lower uptake in all normal organs and tissues except from the stomach, muscles, and bones. The most pronounced was the blocking effect in the salivary gland, lungs, spleen, pancreas, and kidneys (Table 1). The blocking of the uptake in kidneys was more efficient (*p* < 0.005, one-way ANOVA) when unlabeled BQ0413 was used (renal uptake, 12 ± 2 %IA/g) in comparison with when PSMA-11 was used (renal uptake, 57 ± 2 %IA/g). It should be mentioned that there was a tendency toward decrease in tumor uptake in the blocking groups: 38 ± 6 %IA/g in the [^99m^Tc]Tc-BQ0413 (40 pmol) group, 31 ± 4 %IA/g in the [^99m^Tc]Tc-BQ0413 (40 pmol) + PSMA-11 (5 nmol) group, and 33 ± 3 %IA/g in the [^99m^Tc]Tc-BQ0413 (40 pmol) + BQ0413 (5 nmol) group, but the difference was not significant.

The effect of co-injected unlabeled tracers on tumor-to-organ ratios (T/O) is presented in Table 2. T/O ratios were higher for all organs in the [^99m^Tc]Tc-BQ0413 (40 pmol) + PSMA-11 (5 nmol) and [^99m^Tc]Tc-BQ0413 (40 pmol) + BQ0413 (5 nmol) groups compared with the [^99m^Tc]Tc-BQ0413 (40 pmol) group, notably for the salivary glands, lungs, spleen, pancreas, and kidneys (*p* < 0.05). The tumor-to-liver ratio significantly (*p* < 0.05) increased in the [^99m^Tc]Tc-BQ0413 (40 pmol) + BQ0413 (5 nmol) group. The tumor-to-muscle ratio significantly (*p* < 0.05) increased in the [^99m^Tc]Tc-BQ0413 (40 pmol) + PSMA-11 (5 nmol) group.

**Figure 8:**
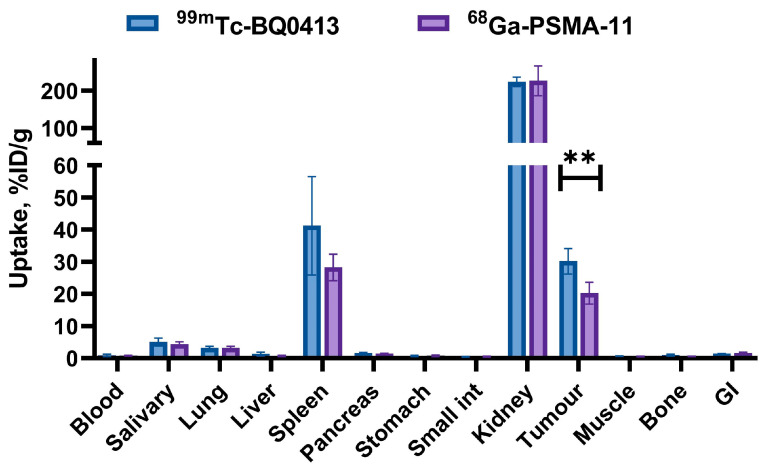
In vivo targeting properties of [^99m^Tc]Tc-BQ0413 (40 pmol/mouse) and [^68^Ga]Ga-PSMA-11 (40 pmol/mouse) in PC3-pip tumor-bearing mice at 1 h post-injection. ** indicates a *p*-value less than 0.05 in a 2-tailed *t*-test. The data are presented as the average (n = 4) and SD.

A comparison of the biodistribution profile and the targeting properties of [^99m^Tc]Tc-BQ0413 (40 pmol/mouse) with the ^68^Ga-labeled PSMA analog PSMA-11 (40 pmol/mouse) at 3 h pi (Figure 8) (details in Table A4 Appendix A) demonstrated that the tumor uptake of [^99m^Tc]Tc-BQ0413 (30 ± 4 %IA/g) was significantly higher (*p* < 0.05, unpaired *t*-test) than the tumor uptake of [^68^Ga]Ga-PSMA-11 (20 ± 3 %IA/g).

**Figure 9:**
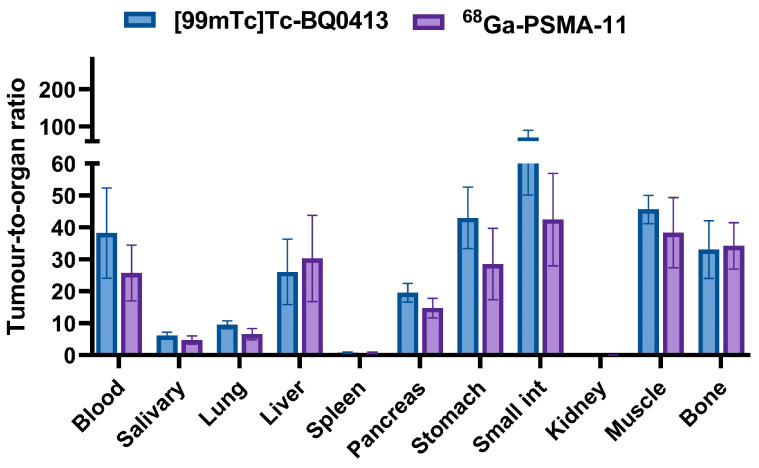
Comparison of tumor-to-organ ratios of [^99m^Tc]Tc-BQ0413 and [^68^Ga]Ga-PSMA-11 in PC3-pip tumor-bearing mice at 1 h post-injection. The data are presented as the average (n = 4) and SD.

A comparison of tumor-to-organ ratios (T/O) 4 h after an injection of [^99m^Tc]Tc-BQ0413 (40 pmol/mouse) and [^68^Ga]Ga-PSMA-11 (40 pmol/mouse) is presented in Figure 9 (details in Table A5 of Appendix A). After an injection of 40 pmol/mouse of [^99m^Tc]Tc-BQ0413, T/kidney and T/lung ratios were significantly higher compared with ratios for the [^68^Ga]Ga-PSMA-11 (40 pmol/mouse) group (*p* < 0.05). Additionally, there was a tendency, although not statistically significant, toward an increase in T/blood, T/salivary gland, T/pancreas, T/muscle, T/stomach, T/small intestine ratios in the [^99m^Tc]Tc-BQ0413 group.

**Figure 10:**
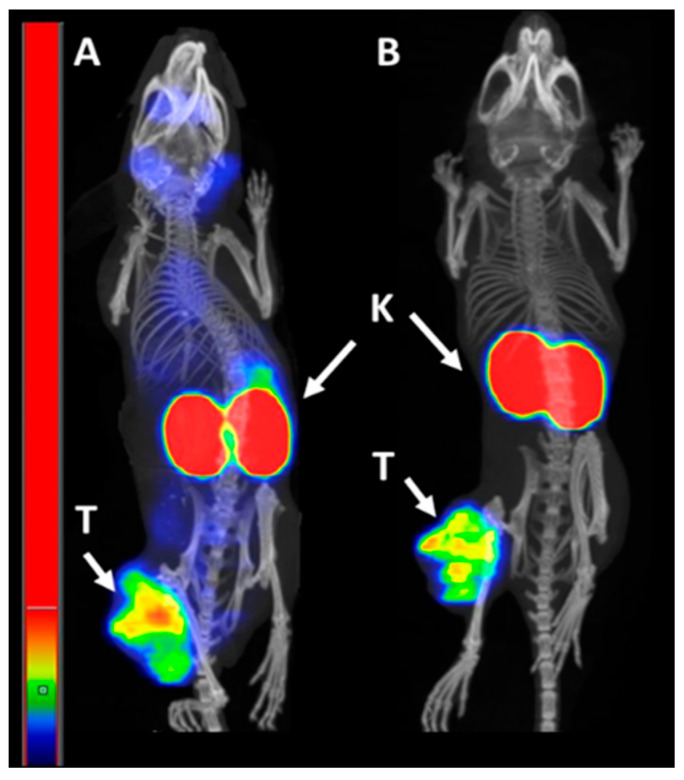
Representative nanoScan SPECT/CT images of PC3-pip xenografted mice injected with [^99m^Tc]Tc-BQ0413 (40 pmol, 1 MBq) at 1 h pi (**A**) and 3 h pi (**B**). Arrows point at tumors (T) and kidneys (K). The linear scale was adjusted to the first red pixel in the tumor.

NanoScan SPECT/CT images of mice injected with [^99m^Tc]Tc-BQ0413 (Figure 10) confirmed the results from the ex vivo biodistribution measurements. High-activity uptake could be visualized in kidneys and tumors both at 1 and 3 h pi. The imaging contrast increased at 3 h pi.

**Figure 11:**
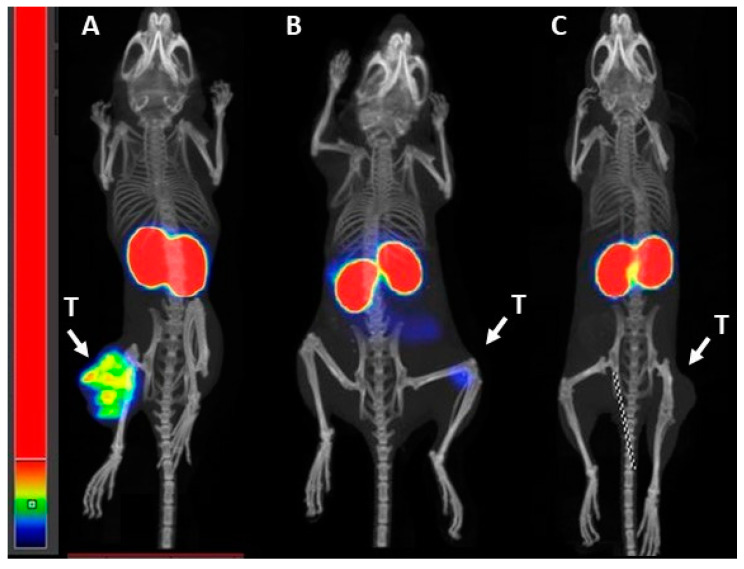
Representative nanoScan SPECT/CT images of PC3-pip xenograft (**A**) compared with PSMA-negative PC-3 (**B**) and SKOV-3 (**C**) xenografts after injection with [^99m^Tc]Tc-BQ0413 (40 pmol, 1 MBq) at 3 h pi. Arrows point at tumors (T).

The uptake of [^99m^Tc]Tc-BQ0413 in PSMA-negative SKOV-3 ovarian carcinoma xenografts and PC-3 prostate carcinoma xenografts was apparently lower than in PC3-pip xenografts in images produced by nanoScan SPECT/CT at 3 h pi, which confirmed the results of the in vivo binding specificity test (Figure 11).

**Figure 12:**
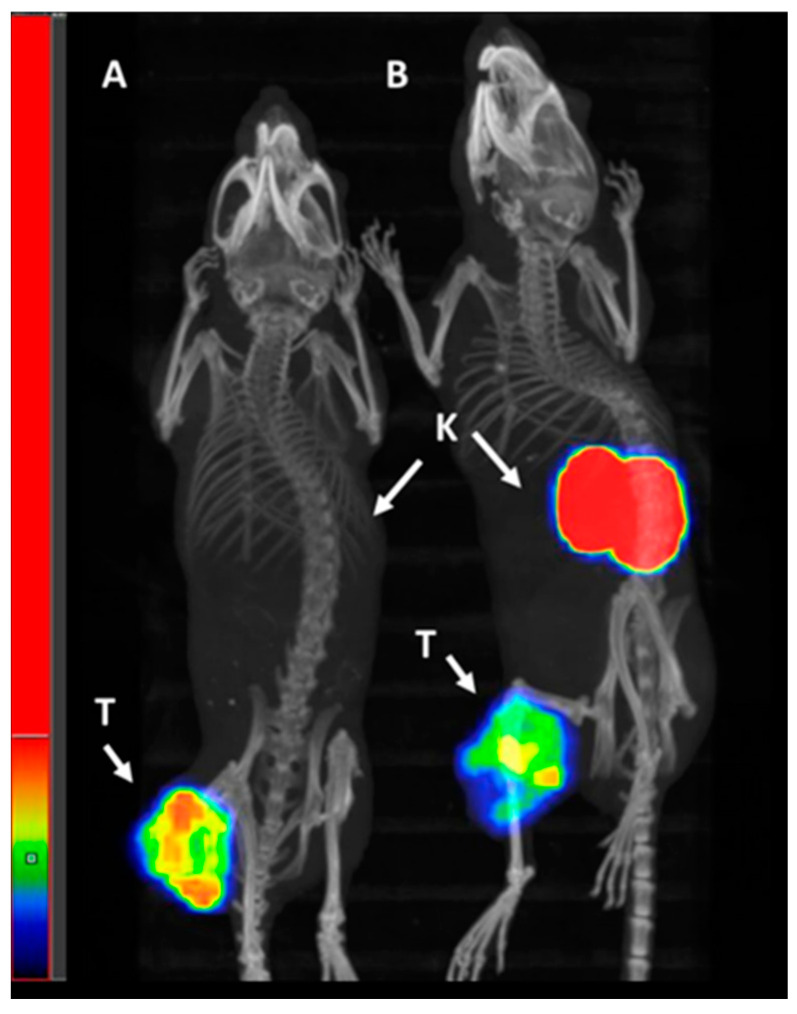
Representative nanoScan SPECT/CT images of PC3-pip xenografted mice injected with 40 pmol/mouse and 1 MBq of [^99m^Tc]Tc-BQ0413 (**B**) and co-injected with 5 nmol/mouse of unlabeled BQ0413 (**A**) at 3 h pi. Arrows point at tumors (T) and kidneys (K).

SPECT/CT imaging demonstrated that a co-injection of 40 pmol/mouse of [^99m^Tc]Tc-BQ0413 with 5 nmol/animal of unlabeled BQ0413 resulted in a visible reduction in renal uptake without a reduction in tumor uptake compared with animals injected with 40 pmol/mouse of [^99m^Tc]Tc-BQ0413 (Figure 12).

**Figure 13:**
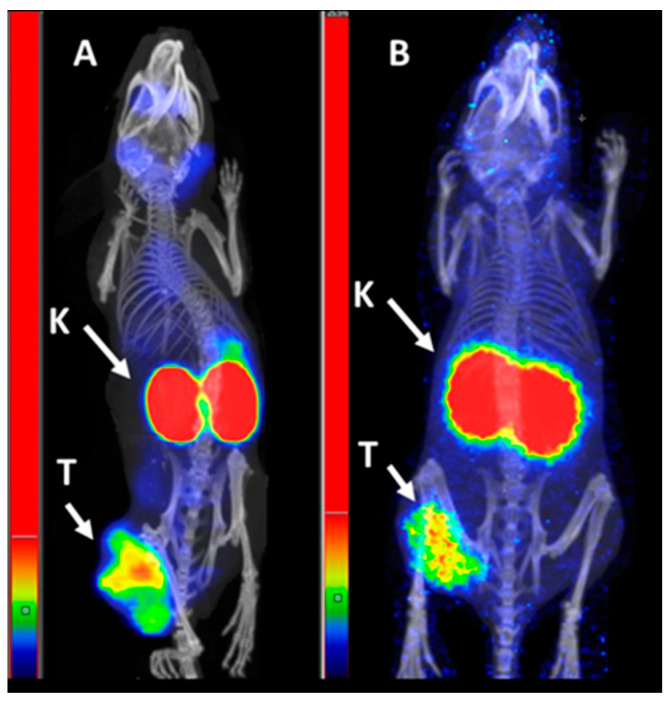
Single-photon emission computed tomography/computed tomography (SPECT/CT) image of PC3-pip xenografted mouse injected with 40 pmol of 1 MBq [^99m^Tc]Tc-BQ0413 at 1 h pi (**A**) and positron emission tomography/computed tomography (PET/CT) of PC3-pip xenografted mouse injected with 40 pmol of 1 MBq [^68^Ga]Ga-PSMA-11 at 1 h pi (**B**). Arrows point at tumors (T) and kidneys (K).

The results from the head-to-head comparison of [^68^Ga]Ga-PSMA-11 and [^99m^Tc]Tc-BQ0413 were proven via positron emission tomography/computed tomography (PET/CT) and single-photon emission computed tomography/computed tomography (SPECT/CT), respectively (Figure 13). The contrast in tumor imaging provided by [^99m^Tc]Tc-BQ0413 was (at least) not inferior to the contrast provided by [^68^Ga]Ga-PSMA-11.

To evaluate the dosimetry, the absorbed dose for men in each organ (Table 3) was estimated based on the ex vivo data from mice (Table A6 Appendix A) using Equation (1). The organ with the highest absorbed dose should be the kidneys (0.1170 mGy/MBq). Doses in other organs and tissues were expected to be below 10^−2^ mGy/MBq. The estimated effective dose for humans was 0.0018 mSv/MBq.

## 3. Discussion

A huge increase in the development of new agents targeting PSMA for theranostic purposes has been observed during the last decade. Imaging agents targeting PSMA have been developed for the SPECT and PET platforms. The most extensively investigated type of high-affinity inhibitors are urea-based ligands. Clinical practice demonstrates the good specificity of such tracers labeled with positron emitters [9]. Current progress in the development of SPECT/CT cameras creates a potential to use this modality for the detection and staging of prostate cancer. Radionuclide molecular imaging using SPECT/CT is an accessible multimodal technology with high quantification accuracy. It enables the visualization of multiple metastases addressing the heterogeneity of expression. The use of radioligands with the ability to bind to PSMA makes molecular imaging a promising approach for both the diagnosis of prostate cancer and the selection of patients for PSMA-targeting radionuclide therapy.

Herein, we present the results of a preclinical evaluation of a novel PSMA-targeting radioligand, [^99m^Tc]Tc-BQ0413, for the SPECT/CT imaging of prostate cancer. The labeling of BQ0413 with Tc-99m using a pre-formulated kit approach provided high radiochemical yield and purity, and the tracer could be injected without a purification step. The targeting properties of [^99m^Tc]Tc-BQ0413 were further investigated in vitro and in vivo using a comprehensive methodology [22,23]. In vitro data show that [^99m^Tc]Tc-BQ0413 binds specifically to PSMA-expressing cells since the presaturation of the target with unlabeled PSMA-11 leads to a significant decrease in activity uptake (Figure 2). Moreover, the binding of [^99m^Tc]Tc-BQ0413 to PSMA-transfected PC3-pip cells was significantly higher compared with binding to PSMA-negative PC-3 cells (Figure 2). [^99m^Tc]Tc-BQ0413 bound to PSMA in PC3-pip cells with picomolar affinity (K_D_ = 33 ± 15 pM) (Figure 3 and Figure 4). The in vitro binding kinetics demonstrated an extremely slow off-rate. These results suggest that the affinity of [^99m^Tc]Tc-BQ0413 to PSMA is higher than the reported affinity of clinically used PSMA inhibitors (Table 4). Our data show that the chemical nature of the radionuclide–chelator moiety plays a substantial role in the binding affinity. The use of the same measurement methodology demonstrated that the affinity of homologous [^111^In]In-BQ7869 with the NOTA chelator was 6.5 ± 0.9 nM [16]. The binding of [^177^Lu]Lu-BQ7876 (DOTA chelator) to PC3-pip cells had two types of interactions: a stronger one (K_D1_ = 3.8 nM) and a weaker one (K_D2_ = 25 nM) [17]. Thus, the use of the [^99m^Tc]Tc-maE3 complex provided the highest affinity in a series of ligands with the same linker.

The biodistribution pattern after an injection of 40 pmol showed high-activity uptake in the tumors (Figure 5 and Figure 10). The accumulation of [^99m^Tc]Tc-BQ0413 in PSMA-positive PC3-pip xenografts was significantly higher than the accumulation in control PSMA-negative PC-3 and SKOV-3 xenografts (Figure 7 and Figure 11), which shows that the tracer’s tumor uptake was dependent on PSMA expression, i.e., target-specific. A noticeable uptake was also observed in the kidneys, spleen, salivary gland, and lungs. The elevated uptake in these organs was expected given the physiological PSMA expression, which has been reported for other PSMA-targeting agents [28,29]. The uptake of activity in the remaining organs at 1 h pi was approximately 1%IA/g or below (Figure 5). The activity uptake decreased over time in the normal organs. These results demonstrate rapid clearance from all organs and tissues (excluding the kidneys). The uptake in the content of the intestines was below 2% of the injected activity at all time points, which indicates that the excretion via bile was not substantial. Thus, the major clearance was through glomerular filtration. This is consistent with the high uptake in kidneys, which amounted to 90% of the injected activity 1 h pi. The retention of activity in the kidneys was consistently high, between 1 and 6 h. Renal uptake might be partially blocked by the addition of an excess amount of PSMA-11 or BQ0413 (Figure 7), which is in agreement with the literature data reporting the expression of PSMA in kidneys [30]. The renal excretion pathway is typical for hydrophilic substances. This confirmed our assumption that the glutamate-based chelator would provide an optimal clearance pattern, permitting the detection of abdominal metastases.

Tumor-to-organ ratios increased from 1 to 6 h pi for all organs, and the highest ratios were T/blood, T/muscle, T/liver, and T/bone, which might be important for the detection of metastases (Figure 6 and Figure 10). An essential factor in successful radionuclide imaging is high imaging contrast because it determines the diagnostic sensitivity [31,32]. Thus, imaging agents that provide high tumor-to-organ ratios (ratios of activity concentration in tumors to concentration in normal organs), especially for organs that are frequent metastatic sites, are required. Metastatic sites in prostate cancer with the most frequent involvement are the bones (90%), lungs (46%), and liver (25%) [33]. In addition, the detection of local lymph node metastases is essential in the early stages of prostate cancer. Thus, sufficiently high tumor-to-bone, tumor-to-lung, tumor-to-liver, and tumor-to-muscle ratios are the preconditions for the successful translation of imaging probes to clinics. In addition, a high tumor-to-blood ratio is an impermeable parameter for the evaluation of an imaging agent, as a blood-borne activity might contribute to the background signal.

Earlier preclinical studies have indicated that it is possible to the saturate uptake of radiolabeled PSMA ligands in normal tissues with a minor (if any) impact on tumor uptake [30,34,35]. This approach is based on the higher expression level of PSMA in tumors (and, accordingly, the higher binding potential of targeting molecules) than in normal tissues. For example, Kalidindi and co-workers demonstrated that the addition of 500 pmol of PSMA-11 to injected [^177^Lu]Lu-PSMA-617 resulted in a significant decrease in uptake in salivary gland and the kidneys but not in tumors [30]. In this study, we evaluated two approaches to blocking normal tissues, the use of unlabeled PSMA-11 (the same approach used by Kalidindi and co-workers) and the use of unlabeled BQ0413. Our data (Table 1 and Figure 12) show that a radical reduction in normal tissue uptake is possible using both approaches even as the tumor uptake remains unaffected. Remarkably, the uptake of [^99m^Tc]Tc-BQ0413 in normal tissues was also significantly reduced by increasing the injected mass of the tracer (Table 1). Most likely, the normal PSMA-expressing tissues acted as a depot for [^99m^Tc]Tc-BQ0413. The dissociation of the tracer from the depot organs resulted in its appearance in blood and normal unPSMA-expressing organs, which were in equilibrium with blood. Thus, the addition of blocking agents resulted in an increase in tumor-to-organ ratios for all organs (Table 2), i.e., increased imaging contrast for all potential metastatic sites. This effect should contribute to better sensitivity in the imaging of small lesions. The use of PSMA-11 for blocking resulted in significantly higher renal uptake, which shows that the use of this PSMA ligand is less efficient in the displacement of [^99m^Tc]Tc-BQ0413 in the kidneys. This is conceivable because the affinity of PSMA-11 for PSMA is worse than the affinity of BQ0413 [27]. These results indicate the possibility of blocking PSMA in normal organs and tissues by increasing the mass of injected [^99m^Tc]Tc-BQ0413 (i.e., a decrease in specific activity of injected peptide-). From a clinical translation point of view, such an approach is preferable to the use of two different agents in the same preparation. A similar effect regarding the positive impact of optimal specific activity on imaging contrast has been observed for other imaging probes, e.g., monoclonal antibody 225 targeting anti-epidermal growth factor receptor [36], scaffold-based protein affibodies, ADAPT and DARPin targeting epidermal growth factor receptor type 2 [37], and small-peptide targeting somatostatin receptor [38]. The important conclusion from this study is that a careful dose-finding study has to be performed during the clinical development of [^99m^Tc]Tc-BQ0413.

A direct head-to-head comparison of [^99m^Tc]Tc-BQ0413 with [^68^Ga]Ga-PSMA-11 in the same model showed 1.5-fold higher tumor uptake for [^99m^Tc]Tc-BQ0413 (Figure 8), which enabled better imaging contrast (Figure 9 and Figure 13). The reduced bone, lung, and hepatobiliary uptake might be favorable for the diagnosis of metastases in prostate cancer.

The dosimetry estimations for [^99m^Tc]Tc-BQ0413 were in low values of mGy/MBq, predicting low absorbed doses in organs and an effective dose of 0.0018 mSv/MBq, as shown in Table 3. The estimated effective dose from the dosimetry study was within the same range as the doses of the majority of other technetium-99m-labeled tracers reported in the literature (Table 5). It is, however, worth noting that some of the reported effective doses in Table 3 are based on clinical patient data, and some are estimated values from preclinical studies. A direct comparison of the values is, therefore, not entirely possible. Significant species differences in the context of activity uptake and retention in the healthy organs of PSMA-targeted tracers between mice and men are generally valid and need further investigation. These differences might reflect both PSMA expression patterns and metabolic activity. Nevertheless, the high kidney uptake of PSMA-targeted probes in mice should not be overvalued and regarded as an exclusion criterion for clinical translation.

Taken together from a biodistribution, in vivo specificity, and radiation dosimetry point of view, [^99m^Tc]Tc-BQ0413 could be a promising candidate for the SPECT/CT imaging of PSMA expression in the clinics. The good match of the technetium-99m half-life with the fast kinetics of peptide-based imaging probes is one reason why [^99m^Tc]Tc-BQ0413 could be a suitable radiotracer for PSMA-targeting. An additional advantage of the technetium-99m label is that it is generator-produced and therefore relatively cheap and easily accessible around the world. Finally, technetium-99m enables SPECT imaging in the hospital, which is more accessible than PET cameras.

## 4. Materials and Methods

BQ0413 (shown in Figure 1) was synthesized by Pepmic Co., Ltd. (Suzhou, China) according to our design. PSMA-11 was purchased from ABX Advanced Biochemical Compounds (Radeberg, Germany). The human prostate cancer cell line PC-3 and the ovarian carcinoma cell line SKOV-3 were purchased from the American Type Culture Collection (ATCC; LGC Promochem, Borås, Sweden). The PSMA-transfected PC3-pip cell line was provided by Prof. Martin G. Pomper, Johns Hopkins University, Baltimore, MD, USA. The cell lines were maintained in RPMI-1640 media (Flow Laboratories, Irvine, UK); PC3-pip cells were maintained with the addition of 10 mg/mL of puromycine (MP Biomedicals, Santa Ana, CA, USA) every second passage. Media supplements (10% fetal bovine serum, penicillin–streptomycin (100 IU/mL penicillin, 100 µg/mL streptomycin), 2 mM L-glutamine, and trypsin-EDTA solution for cell detachment) were purchased from Biochrom AG (Berlin, Germany). Technetium-99m was obtained as [^99m^Tc]NaTcO4 via elution from a ^99^Mo/^99m^Tc generator (Mallinckrodt Inc., St. Louis, MO, USA). The radioactivity content in cells and organs was measured using the 2480 Wizard2TM gamma counter (PerkinElmer, Waltham, MA, USA).

### 4.1. Radiochemistry

#### 4.1.1. BQ0413 ^99m^Tc-Labeling and Stability Control

BQ0413 (10 µg) dissolved in phosphate-buffered saline (PBS) (5 mg/mL) was added to a freeze-dried kit containing 5 mg of gluconic acid sodium salt (Celsus Laboratories, Geel, Belgium), 75 µg of stannous chloride (Fluka Chemika, Buchs, Switzerland), and 100 µg of EDTA (Sigma-Aldrich, Munich, Germany) [45]. Freshly eluted ^99m^Tc-pertechnetate (20–60 MBq per 1 µg, 85 µL) was added to the mixture, and the vial was incubated at 90 °C for 60 min. The radiochemical yield was analyzed using instant thin-layer chromatography (iTLC) strips (Agilent Technologies, Santa Clara, CA, USA) eluted with acetone (R_f_ = 0 for [^99m^Tc]Tc-BQ0413 and [^99m^Tc]Tc-TcO_2_; R_f_ = 1 for [^99m^Tc]Tc-TcO_4_^−^) and PBS (R_f_ = 0 for [^99m^Tc]Tc-TcO_2_; R_f_ = 1 for the radiolabeled tracer). iTLC was analyzed using the Cyclone Plus Storage Phosphor System (PerkinElmer, Waltham, MA, USA). The radiochemical purity was quantified using radio high-performance liquid chromatography (HPLC). Radio-HPLC analysis was performed using a Hitachi Chromaster HPLC system (Hitachi, VWR, Darmstadt, Germany) with a radioactivity detector and a Phenomenex Luna^®^ C18 column (100 Å; 150 × 4.6 mm; 5 µm) at room temperature (20 °C). Solvent A was 0.1% trifluoroacetic acid (TFA) in H_2_O, solvent B was 0.1% TFA in acetonitrile, and the flow rate was 1 mL/min. For identity and purity analysis of [^99m^Tc]Tc-BQ0413, a method with a gradient from 5 to 90% solvent B over 20 min was used. The radio-HPLC chromatograms of radiolabeled compounds can be found in the Supplementary Materials.

To test the stability, the radiolabeled compound was incubated with a 300 × molar excess of cysteine or with PBS at room temperature for 1 h and in human plasma at 37 °C for 1 h.

#### 4.1.2. 68Ga-Labeling of PSMA-11 for Comparative Studies

PSMA-11 (5–10 µg) was diluted in 0.1 M NaOAc buffer with a pH of 4.3. Gallium-68 was eluted from a ^68^Ge/^68^Ga-generator (Eckert & Ziegler Co., Ltd. (Berlin, Germany)) using 0.1 M metal-free hydrochloric acid. Freshly eluted ^68^GaCl_3_ (100–120 MBq) was added to the mixture, and then the vial was incubated at 75 °C for 10 min. The radiochemical yield and purity were determined using radio-HPLC analysis; the gradient schedule was as follows: 15% to 45% B, 5 min; 45% to 90%, 0.5 min; 90% B for 2 min; 90% to 15% B over 0.5 min, and remaining 15% B for 2 min. Because of high radiochemical purity, [^68^Ga]Ga-PSMA-11 was used without further purification, stabilized by adding a 300 × molar excess of Na_2_EDTA. The radio-HPLC chromatogram of [^68^Ga]Ga-PSMA-11 can be found in the Supplementary Materials (Figure S2 in Supplementary Material).

### 4.2. In Vitro Characterization

In vitro Binding Specificity: Approximately 8 × 10^5^ of PC3-pip or PC-3 cells per well were seeded in 6-well plates 24 h before the experiment. To saturate the target, one set of cells was pre-treated with unlabeled PSMA-11 (500 nM per well), while the second set was treated with complete media. After 15 min of incubation at room temperature, ^99m^Tc-labeled BQ0413 was added to all wells to reach a concentration of 2 nM, and cells were incubated at 37 °C. After 1 h, media were aspirated, and cells were treated with trypsin-EDTA and collected. The activity of the detached cells was measured using a gamma counter.

Affinity Measurements: The binding kinetics of ^99m^Tc-labeled BQ0413 were measured in real time using LigandTracer Yellow Instruments (Ridgeview Instruments AB, Uppsala, Sweden) on PC3-pip cells at room temperature. The uptake was recorded after adding 5 nM of the radiolabeled BQ0413 for 90 min. After measuring the uptake kinetics, the medium containing labeled BQ0413 was replaced with fresh medium, and the dissociation was monitored over 7 h. The association rate (k_a_) and the dissociation rate (k_d_) were computed using a 1:1 kinetic binding model in the TraceDrawer software version 1.9.2 (Ridgeview Instruments AB, Uppsala, Sweden). The equilibrium dissociation constant, K_D_, was calculated by dividing k_d_ with k_a_.

Cellular Processing: To evaluate the cellular processing of the ^99m^Tc-labeled BQ0413, PC3-pip cells were incubated with the radiolabeled tracer (2 nM), and at predetermined time points (1, 2, 4 and 8 h), the membrane-bound and internalized fractions were collected as previously described using a 4 M urea solution in 0.2 M glycine buffer (pH 2) to collect the membrane-bound fraction and the 1 N solution of NaOH and to collect the internalized fraction [46]. Samples were measured with the gamma counter to determine activity content.

### 4.3. In Vivo Assays

All in vivo experiments were carried out on BALB/c nu/nu or NMRI mice purchased from Scanbur A/S (Sollentuna, Sweden). All animal studies were approved by the Ethics Committee for Animal Research in Uppsala (Sweden), following the national legislation on the protection of laboratory animals (permit 5.8.18-00473/2021, approved 26 February 2021). Prostate cancer xenografts expressing PSMA were established in BALB/c nu/nu mice via subcutaneous injection of PC3-pip cell suspension in PBS (10^7^ cells/animal in 100 µL). For in vivo specificity control, 10^7^ cells/animal PSMA-negative SKOV-3 or PC-3 cells were implanted. Average animal weight was 18.3 ± 1.2 g. Average tumor weights were 0.13 ± 0.06 g, 0.18 ± 0.08 g, and 0.12 ± 0.09 g for PC3-pip, SKOV-3, and PC-3 xenografts, respectively. Four mice per data point were used in in vivo experiments; two mice per data point were used in SPECT/CT (^99m^Tc-labeled BQ0413) and PET/CT (^68^Ga-labeled PSMA-11) imaging experiments.

#### 4.3.1. Biodistribution in Tumor-Bearing Mice

Each mouse was intravenously injected with 40 pmol of [^99m^Tc]Tc-BQ0413 (60 kBq; mass of injected compound was adjusted with unlabeled tracer). The mice were euthanized after lethal intraperitoneal injection of ketamine/xylazine followed by exsanguination 1, 3, and 6 h pi. The organs of interest were collected, weighed, and measured for their activity content using the gamma counter. The uptake activity in organs was calculated as the percentage of injected activity per gram (%IA/g) for blood, salivary gland, lungs, liver, spleen, pancreas, stomach, small intestine, kidneys, tumor, muscle, and bone tissue and as %IA for the remaining carcass and the rest of the intestines with content.

#### 4.3.2. Binding Specificity to PSMA

The in vivo specificity of [^99m^Tc]Tc-BQ0413 was investigated using 2 control groups: (a) PSMA-negative PC-3 (prostate carcinoma) xenografts; (b) PSMA-negative SKOV-3 (ovarian carcinoma) xenografts. Each mouse was intravenously injected with 40 pmol of [^99m^Tc]Tc-BQ0413 (60 kBq; mass of injected tracer was adjusted with unlabeled compound). Animals were euthanized, organs were collected, and uptake activity was measured according to the protocol described above.

#### 4.3.3. Efficacy of Blocking Normal Tissue Uptake

The experiment was performed to evaluate the efficacy of blocking normal tissue uptake using a co-injection of an additional mass of BQ0413 or non-labeled PSMA-11. Mice bearing PC3-pip xenografts were injected with a mixture of 40 pmol of [^99m^Tc]Tc-BQ0413 with 5 nmol of unlabeled BQ0413 or PSMA-11 per animal. Animals were euthanized at 3 h pi, organs were collected, and activity was measured according to the protocol described above.

#### 4.3.4. Comparison of Targeting Properties of [^99m^Tc]Tc-BQ0413 and [^68^Ga]Ga-PSMA-11

In order to compare the biodistribution profile and the targeting properties of [^99m^Tc]Tc-BQ0413 with the ^68^Ga-labeled PSMA analog PSMA-11, each mouse was intravenously injected with 40 pmol of radiotracer (60 kBq; mass of injected tracer was adjusted with unlabeled tracer). Animals were euthanized, organs were collected, and activity uptake was measured according to the protocol described above.

#### 4.3.5. Imaging

The mice were intravenously injected with 40 pmol (1 MBq) of [^99m^Tc]Tc-BQ0413. Whole-body SPECT/CT scans were performed at 1 and 3 h pi using nanoScan SPECT/CT (Mediso Medical Imaging Systems Ltd., Budapest, Hungary). SPECT raw data were reconstructed using Tera-Tomo™ 3D SPECT reconstruction technology (version 3.00.020.000; Mediso Medical Imaging Systems Ltd., Budapest, Hungary): normal dynamic range; 30 iterations; and 1 subset. CT data were reconstructed using Filter Back Projection in the Nucline 2.03 software (Mediso Medical Imaging Systems Ltd., Budapest, Hungary). SPECT and CT files were fused using the Nucline 2.03 software and are presented as maximum intensity projections in the RGB color scale.

For the whole-body PET/CT imaging, mice were intravenously injected with 40 pmol (1 MBq) of [^68^Ga]Ga-PSMA-11. The imaging procedure was performed at 1 h pi using a nanoScan PET/MRI 3T camera (Mediso Medical Imaging Systems Ltd., Budapest, Hungary). Immediately after PET scan, a CT scan was acquired using a nanoScan SPECT/CT camera (Mediso Medical Imaging Systems Ltd., Budapest, Hungary) with the same bed. Reconstruction of the PET/CT scans was conducted using the Nucline nanoScan 3.04.014.0000 software.

#### 4.3.6. Dosimetry Estimation

To estimate the organ-absorbed doses after intravenous injection of [^99m^Tc]Tc-BQ0413, NMRI mice were injected with 40 pmol (60–650 kBq) of the radiolabeled tracer, and biodistribution was evaluated 1, 3, 6, and 24 h pi. Sample collection and activity measurements were performed as described above. To evaluate dosimetry in humans, uptake values in mice were upscaled using the well-established “percent kg/g method” [37] (Equation (1)):

(%IA/organ)human = [(%IA/g)animal × (kg TBweight)animal × (g organ)/(kg TBweight)human],(1)

where %IA-% is the injected activity.

Organ uptakes for humans were calculated using organ weights of the reference adult male (ICRP publication 23). The data were fitted using a single exponential function, and residence time was calculated as an area under a fitted curve using the GraphPad Prism software version 10.1.0 (316) for Windows (GraphPad Software, San Diego, CA, USA). Absorbed doses were calculated using OLINDA/EXM 1.1 for Adult Male phantoms.

### 4.4. Data Analysis

Obtained values are presented as averages with standard deviations. Statistical treatment and linear regression analysis were performed using GraphPad Prism software version 10.1.0 (316) for Windows (GraphPad Software, San Diego, CA, USA). The difference was considered significant when the *p*-value was less than 0.05.

## 5. Conclusions

We developed a novel agent for SPECT imaging PSMA expression. BQ0413 was labeled with technetium-99m with high stability through single-step labeling. [^99m^Tc]Tc-BQ0413 bound specifically to PSMA-expressing cells with exceptionally high affinity in a low picomolar range. In vivo studies demonstrated high and target-specific uptake of [^99m^Tc]Tc-BQ0413 in PSMA-overexpressing human xenografts in mice. We demonstrated, in a murine model, that the imaging contrast could be improved by varying the mass of the injected peptide. The dosimetry for a single administration of [^99m^Tc]Tc-BQ0413 is favorable for a clinical translation. Special attention should be paid to finding an optimal injected mass for [^99m^Tc]Tc-BQ0413 during clinical evaluation.

## Figures and Tables

**Table 1:** Efficacy of blocking [^99m^Tc]Tc-BQ0413 uptake in normal PSMA-expressing tissues evaluated with a co-injection of 40 pmol of [^99m^Tc]Tc-BQ0413 with 5 nmol/animal of unlabeled BQ0413 or 5 nmol/animal of unlabeled PSMA-11. Data are expressed as the percentage of administered activity (injected probe) per gram of tissue (%IA/g). The data are presented as the average (n = 4) and SD.

Organ	[^99m^Tc]Tc-BQ0413 (40 pmol)	[^99m^Tc]Tc-BQ0413 (40 pmol) + BQ0413 (5 nmol)	[^99m^Tc]Tc-BQ0413 (40 pmol) + PSMA-11 (5 nmol)
Blood	0.32 ± 0.05 ^a^	0.11 ± 0.02	0.13 ± 0.03
Salivary gland	2.3 ± 0.3 ^a,b^	0.27 ± 0.07	0.3 ± 0.07
Lungs	1.8 ±0.3 ^a,b^	0.25 ± 0.06	0.24 ± 0.04
Liver	0.43 ± 0.07 ^a,b^	0.15 ± 0.04	0.21 ± 0.06
Spleen	13 ± 3 ^a,b^	1.0 ± 0.2	0.85 ± 0.05
Pancreas	1.1 ± 0.2 ^a,b^	0.15 ± 0.04	0.11 ± 0.02
Stomach	0.5 ± 0.1	0.3 ± 0.1	0.2 ± 0.1
Small intestine	0.3 ± 0.1 ^b^	0.11 ± 0.09	0.19 ± 0.03
Kidneys	241 ± 21 ^a,b^	12 ± 2 ^c^	57 ± 2
Tumor	38 ± 6	33 ± 3	31 ± 4
Muscle	0.3 ± 0.1	0.06 ± 0.02	0.07 ± 0.02
Bone	0.5 ± 0.1	0.4 ± 0.2	0.5 ± 0.1
Intestines with content *	2.0 ± 0.2	1.7 ± 0.2	1.7 ± 0.1
Carcass *	8.4 ± 0.7	2.3 ± 0.9	2 ± 2

^a^ Significant difference (*p* < 0.05) between [^99m^Tc]Tc-BQ0413 (40 pmol) and [^99m^Tc]Tc-BQ0413 (40 pmol) + BQ0413 (5 nmol); ^b^ significant difference (*p* < 0.05) between [^99m^Tc]Tc-BQ0413 (40 pmol) [^99m^Tc]Tc-BQ0413 (40 pmol) + PSMA-11 (5 nmol); ^c^ significant difference (*p* < 0.05) between [^99m^Tc]Tc-BQ0413 (40 pmol) + BQ0413 (5 nmol) and [^99m^Tc]Tc-BQ0413 (40 pmol) + PSMA-11 (5 nmol). ANOVA test (Bonferroni’s multiple comparisons test) was performed to test significant (*p* < 0.05) differences. * Data for intestines with content and carcass are presented as % of injected dose per sample, excluding a small segment of the small intestine.

**Table 2:** Tumor-to-organ ratios after blocking of [^99m^Tc]Tc-BQ0413 uptake in normal PSMA-expressing tissues. The data are presented as the average (n = 4) and SD.

Organ	[^99m^Tc]Tc-BQ0413 (40 pmol)	[^99m^Tc]Tc-BQ0413 (40 pmol) + BQ0413 (5 nmol)	[^99m^Tc]Tc-BQ0413 (40 pmol) + PSMA-11 (5 nmol)
Blood	123 ± 25 ^a^	314 ± 73	253 ± 48
Salivary gland	16 ± 2 ^a,b^	127 ± 20	106 ± 25
Lungs	21 ± 4 ^a,b^	134 ± 30	133 ± 21
Liver	85 ± 5 ^a^	222 ± 55	159 ± 41
Spleen	2.8 ± 0.5 ^a,b^	35 ± 6	37 ± 5
Pancreas	39 ± 9 ^a,b^	235 ± 50	293 ± 45
Stomach	69 ± 6	146 ± 73	171 ± 93
Small intestine	106 ± 20	393 ± 191	170 ± 47
Kidneys	0.20 ± 0.02 ^a,b^	2.8 ± 0.4 ^c^	0.5 ± 0.1
Muscle	123 ± 36 ^b^	539 ± 165	469 ± 103
Bone	67 ± 11	80 ± 28	74 ± 24

^a^ Significant difference (*p* < 0.05) between [^99m^Tc]Tc-BQ0413 (40 pmol) and [^99m^Tc]Tc-BQ0413 (40 pmol) + BQ0413 (5 nmol); ^b^ significant difference (*p* < 0.05) between [^99m^Tc]Tc-BQ0413 (40 pmol) [^99m^Tc]Tc-BQ0413 (40 pmol) + PSMA-11 (5 nmol); ^c^ significant difference (*p* < 0.05) between [^99m^Tc]Tc-BQ0413 (40 pmol) + BQ0413 (5 nmol) and [^99m^Tc]Tc-BQ0413 (40 pmol) + PSMA-11 (5 nmol). ANOVA test (Bonferroni’s multiple comparisons test) was performed to test significant (*p* < 0.05) differences.

**Table 3:** The absorbed dose (mGy/MBq) of [^99m^Tc]Tc-BQ0413 in each of the targeted organs.

Targeted Organ	Absorbed Dose (mGy/MBq)
Adrenals	0.0069
Brain	0.0004
Gallbladder wall	0.0041
Lower large intestine wall	0.0010
Small intestine wall	0.0024
Stomach wall	0.0028
Upper large intestine LI wall	0.0024
Heart wall	0.0017
Kidneys	0.1170
Liver	0.0030
Lungs	0.0012
Muscle	0.0013
Pancreas	0.0051
Red bone marrow	0.0019
Osteogenic cells	0.0024
Skin	0.0006
Spleen	0.0068
Testes	0.0004
Thymus	0.0007
Thyroid	0.0005
Urinary bladder	0.0007
Uterus	0.0011
Total body	0.0018
Effective dose equivalent (mSv/MBq)	0.0092
Effective dose (mSv/MBq)	0.0018

**Table 4:** Equilibrium dissociation constant (K_D_) of some PSMA-targeting tracers.

Tracer	Equilibrium Dissociation Constant, K_D_	Reference
[^177^Lu]Lu-PSMA-617	0.24 nM	[24]
[^18^F]F-DCFPyL	0.49 ± 0.04 nM	[25]
Al [^18^F]F-PSMA-BCH	2.9 ± 0.8 nM	[26]
[^18^F]F-PSMA-11	3.0 ± 0.9 nM	[27]
[^68^Ga]Ga-PSMA-11	0.5 ± 0.2 nM	[27]
[^99m^Tc]Tc-PSMA-T1 [^99m^Tc]Tc-PSMA-T4	11.4 nM	[18]
[^99m^Tc]Tc-MIP-1427 [^99m^Tc]Tc-MIP-1404 [^99m^Tc]Tc-MIP-1428 [^99m^Tc]Tc-MIP-1405	0.6 ± 0.4 nM 1.1 ± 0.9 nM 1.8 ± 0.3 nM 4.3 ± 0.3 nM	[11]

**Table 5:** Effective dose (mSv/MBq) of ^99m^Tc-labeled radiotracers reported in the literature.

Tracer	Effective Dose (mSv/MBq)	Reference
[^99m^Tc]Tc-PSMA I&S	0.0055 *	[20]
[^99m^Tc]Tc-HYNIC-PSMA	0.0003 *	[39]
[^99m^Tc]Tc-HYNIC-PSMA-XL-2	0.0048 *	[40]
[^99m^Tc]Tc-maSSS-PEG2-RM26	0.0035 ^#^	[41]
[^99m^Tc]Tc-ADAPT6	0.009 *	[42]
[^99m^Tc]Tc-DTPATc-DTPA	0.0175 *	[43]
[^99m^Tc]Tc-NM-02	0.0065 *	[44]

* Based on clinical patient data; **^#^** estimated values from preclinical studies.

## Data Availability

The data generated during the current study are available from the corresponding authors upon reasonable request.

## Supplementary Materials

The following supporting information can be downloaded at https://www.mdpi.com/article/10.3390/ijms242417391/s1.

## Appendix A

**Table A1:** In vivo biodistribution of [^99m^Tc]Tc-BQ0413 (40 pmol/mouse) in PC3-pip tumor-bearing mice at 1, 3, and 6 h post-injection showing uptake activity in specified organs. Data are expressed as the percentage of administered activity per gram of tissue (%IA/g). The data are presented as the average (n = 4) and SD.

Organ	1 h	3 h	6 h
Blood	0.9 ± 0.4	0.32 ± 0.05	0.16 ± 0.02
Salivary gland	5 ± 1	2.3 ± 0.3	1.1 ± 0.2
Lungs	3.2 ± 0.5	1.8 ± 0.3	1.5 ± 0.3
Liver	1.3 ± 0.6	0.43 ± 0.07	0.26 ± 0.03
Spleen	41 ± 15	13 ± 3	10 ± 6
Pancreas	1.6 ± 0.3	1.1 ± 0.2	0.5 ± 0.2
Stomach	0.7 ± 0.2	0.5 ± 0.1	0.29 ± 0.1
Small intestine	0.5 ± 0.1	0.3 ± 0.1	0.14 ± 0.02
Kidneys	224 ± 12	241 ± 21	251 ± 21
Tumor	30 ± 4	38 ± 7	39 ± 5
Muscle	0.66 ± 0.08	0.3 ± 0.1	0.17 ± 0.03
Bone	1.0 ± 0.3	0.5 ± 0.1	0.31 ± 0.03
GI tract	1.4 ± 0.1	2.0 ± 0.2	1.5 ± 0.4

**Table A2:** In vivo biodistribution of [^99m^Tc]Tc-BQ0413 (40 pmol/mouse) in PC3-pip tumor-bearing mice at 1, 3, and 6 h post-injection showing tumor-to-organ ratios. The data are presented as the average (n = 4) and SD.

Organ	1 h	3 h	6 h
Blood	38 ± 14	123 ± 25	243 ± 27
Salivary gland	6 ± 1	16 ± 2	35 ± 4
Lungs	10 ± 1	21 ± 4	27 ± 4
Liver	26 ± 10	85 ± 5	152 ± 15
Spleen	0.8 ± 0.2	2.8 ± 0.5	3.5 ± 0.7
Pancreas	20 ± 3	389 ± 9	87 ± 45
Stomach	43 ± 10	69 ± 6	144 ± 31
Small intestine	70 ± 20	106 ± 20	272 ± 24
Kidneys	0.13 ± 0.01	0.17 ± 0.02	0.16 ± 0.02
Muscle	46 ± 4	123 ± 36	236 ± 25
Bone	331 ± 9	87 ± 41	128 ± 24

**Table A3:** Comparison of [^99m^Tc]Tc-BQ0413 (40 pmol/mouse) accumulation in PSMA-positive PC3-pip and PSMA-negative PC-3 and SKOV-3 tumor-bearing mice at 3 h post-injection. Data are expressed as the percentage of administered activity per gram of tissue (%IA/g). The data are presented as the average (n = 4) and SD.

Organ	PC3-pip (PSMA+)	PC-3 (PSMA−)	SKOV-3 (PSMA−)
Blood	0.32 ± 0.05	0.5 ± 0.3	0.38 ± 0.08
Salivary gland	2.3 ± 0.3	3.7 ± 0.9	3.1 ± 0.8
Lungs	1.8 ± 0.3	2.3 ± 0.4	2.5 ± 0.2
Liver	0.43 ± 0.07	1.0 ± 0.4	0.6 ± 0.1
Spleen	13 ± 3	24 ± 11	19 ± 7
Pancreas	1.1 ± 0.2	1.5 ± 0.9	0.9 ± 0.4
Stomach	0.5 ± 0.1	2 ± 2	0.6 ± 0.1
Small intestine	0.3 ± 0.1	0.5 ± 0.3	0.5 ± 0.3
Kidneys	241 ± 21	254 ± 25	300 ± 49
Tumor	38 ± 6	3 ± 2	0.9 ± 0.3
Muscle	0.3 ± 0.1	0.38 ± 0.08	0.4 ± 0.1
Bone	0.5 ± 0.1	0.5 ± 0.2	0.7 ± 0.3
GI tract	2.0 ± 0.2	2.2 ± 0.6	2.1 ± 0.4

**Table A4:** In vivo targeting properties of [^99m^Tc]Tc-BQ0413 (40 pmol/mouse) and [^68^Ga]Ga-PSMA-11 (40 pmol/mouse) in PC3-pip tumor-bearing mice at 1 h post-injection. Data are expressed as the percentage of administered activity per gram of tissue (%IA/g). The data are presented as the average (n = 4) and SD.

Organ	[^99m^Tc]Tc-BQ0413	^[68^Ga]Ga-PSMA-11
Blood	0.9 ± 0.4	0.8 ± 0.1
Salivary gland	5 ± 1	4.4 ± 0.8
Lungs	3.2 ± 0.5	3.2 ± 0.6
Liver	1.3 ± 0.6	0.7 ± 0.2
Spleen	41 ± 15	28 ± 4
Pancreas	1.6 ± 0.2	1.3 ± 0.2
Stomach	0.7 ± 0.2	0.8 ± 0.3
Small intestine	0.5 ± 0.1	0.5 ± 0.1
Kidneys	224 ± 12	226 ± 40
Tumor	30 ± 4	20 ± 3
Muscle	0.66 ± 0.08	0.54 ± 0.08
Bone	1.0 ± 0.3	0.60 ± 0.06
GI tract	1.4 ± 0.1	1.6 ± 0.3

**Table A5:** Comparison of tumor-to-organ ratios of [^99m^Tc]Tc-BQ0413 and [^68^Ga]Ga-PSMA-11 in PC3-pip tumor-bearing mice at 1 h post-injection. The data are presented as the average (n = 4) and SD.

Organ	[^99m^Tc]Tc-BQ0413	[^68^Ga]Ga-PSMA-11
Blood	38 ± 14	26 ± 9
Salivary glands	6 ± 1	5 ± 1
Lungs	10 ± 1	7 ± 2
Liver	26 ± 10	30 ± 14
Spleen	0.8 ± 0.2	0.7 ± 0.2
Pancreas	20 ± 3	15 ± 3
Stomach	43 ± 10	29 ± 11
Small intestine	70 ± 20	42 ± 14
Kidneys	0.13 ± 0.01	0.09 ± 0.02
Muscle	46 ± 4	38 ± 11
Bone	33 ± 9	34 ± 7

**Table A6:** In vivo biodistribution of [^99m^Tc]Tc-BQ0413 (40 pmol/mouse) in NMRI/tac mice at 1, 3, 6, and 24 h post-injection for dosimetry estimation. Data are expressed as the percentage of administered activity per gram of tissue (%IA/g). The data are presented as the average (n = 4) and SD.

Organ	1 h	3 h	6 h	24 h
Blood	0.45 ± 0.10	0.17 ± 0.05	0.09 ± 0.01	0.013 ± 0.004
Heart	0.47 ± 0.08	0.18 ± 0.05	0.09 ± 0.02	0.02 ± 0.01
Lungs	1.4 ± 0.1	0.8 ± 0.1	0.44 ± 0.09	0.04 ± 0.01
Liver	0.37 ± 0.02	0.17 ± 0.08	0.09 ± 0.01	0.014 ± 0.004
Spleen	3 ± 13	1.8 ± 0.4	1.1 ± 0.5	0.09 ± 0.03
Pancreas	0.79 ± 0.03	0.7 ± 0.3	0.38 ± 0.06	0.03 ± 0.01
Kidneys	188 ± 8	228 ± 33	223 ± 23	31 ± 10
Muscle	0.42 ± 0.01	0.16 ± 0.04	0.08 ± 0.01	0.02 ± 0.01
Bone	1.2 ± 0.2	0.58 ± 0.10	0.42 ± 0.07	0.20 ± 0.01
Stomach	0.03 ± 0.01	0.016 ± 0.002	0.012 ± 0.001	0.007 ± 0.001
Small intestine	0.33 ± 0.03	0.4 ± 0.2	0.3 ± 0.1	0.3 ± 0.3
Upper large intestine	1.2 ± 0.2	1.2 ± 0.5	0.7 ± 0.2	0.3 ± 0.2
Large intestine	0.06 ± 0.02	0.17 ± 0.08	0.30 ± 0.06	0.2 ± 0.1
Caecum	0.05 ± 0.01	0.2 ± 0.2	0.20 ± 0.2	0.2 ± 0.1
Carcass	0.11 ± 0.02	0.59 ± 0.07	0.8 ± 0.2	0.5 ± 0.4
