# Supplementary material for: Preclinical Evaluation of a Novel High-Affinity Radioligand [99mTc]Tc-BQ0413 Targeting Prostate-Specific Membrane Antigen (PSMA)
Source: Int J Mol Sci. 2023 Dec 12;24(24):17391. doi: 10.3390/ijms242417391 (PMC10743726; doi:10.3390/ijms242417391)
Supplement: Supplementary file 1 [file ijms-24-17391-s001.zip › ijms-2711517-supplementary.pdf]

Bezverkhniaia E, Kanellopoulos P, Abouzayed A, Larkina M, Oroujeni M, Vorobyeva A, Rosenström U, Tolmachev V, Orlova A.

Preclinical evaluation of a novel high affinity radioligand [^{99m}Tc]Tc-BQ0413 targeting prostate specific membrane antigen (PSMA)

Supplementary Materials

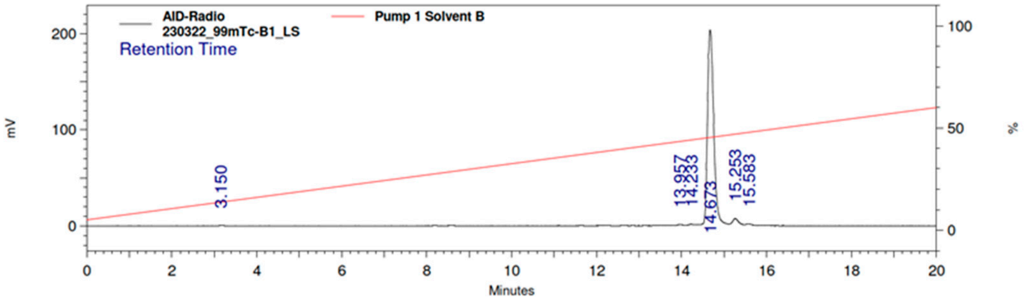

Figure S1. Radio-HPLC chromatogram of [^{99m}Tc]Tc-BQ0413.

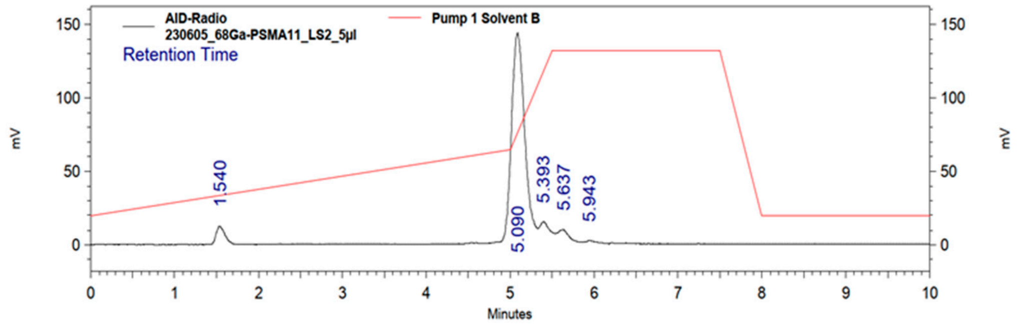

Figure S2. Radio-HPLC chromatogram of [^{68}Ga]Ga-PSMA-11.
